# Saline Injections in Puerperal Eclampsia

**Published:** 1899-07-08

**Authors:** 


### SALINE INJECTIONS IN PUERPERAL ECLAMPSIA.

At the last meeting of the Edinburgh Obstetrical Society Dr. Jardine read a paper giving notes of twelve cases in which he had treated eclampsia by injecting into the subcutaneous tissues considerable quantities of saline solutions. The one which he used was made from a mixture of bicarbonate of potassium one part, and common salt three parts, a drachm of the mixture being added to a pint of sterilised water at a temperature of 100 deg. F. The potash was added because of its diuretic action. An aspirator trocar and cannula, a few feet of tubing, and a piece of adhesive plaster were, he said, all that were needed in the way of apparatus. The whole could be sterilised, and the injection could be made anywhere if the tissues were loose. Under the lax abdominal wall after delivery, or under the edge of the breast before, he had run in a pint in four minutes. Absorption began at once, and the fluid would disappear in from fifteen to twenty minutes. The results had, he thought, been satisfactory.

